# Chronic Aromatase Inhibition Attenuates Synaptic Plasticity in Ovariectomized Mice

**DOI:** 10.1523/ENEURO.0346-24.2024

**Published:** 2024-11-26

**Authors:** Julia Brill, David J. Linden

**Affiliations:** ^1^Solomon H. Snyder Department of Neuroscience, Johns Hopkins University, Baltimore, Maryland 21210; ^2^Kavli Neuroscience Discovery Institute, Johns Hopkins University, Baltimore, Maryland 21210

**Keywords:** cognitive, estradiol, letrozole, neurosteroid, plasticity, synapses

## Abstract

Brain-derived estrogen (17β-estradiol, E2) is a neuromodulator that plays important roles in neural plasticity and network excitability. Chronic inhibition of estrogen synthesis is used in adjuvant breast cancer therapy for estrogen receptor-positive tumors and may have been associated with cognitive and affective side effects. Here, we have developed a model of adjuvant therapy in female ovariectomized mice in which the E2 biosynthetic enzyme aromatase is inhibited by letrozole (1 mg/kg/day, i.p., for up to 3 weeks), Using two-photon longitudinal in vivo imaging in Thy1-GFP-M mice, we found that spine density in the apical dendrites of neocortical layer 5 pyramidal cells was unaffected by letrozole treatment but spine turnover was reduced. LTP in layer 4 to layer 2/3 synapses in the somatosensory cortex was also reduced in slices from letrozole-treated mice, showing deficits in structural and functional plasticity resulting from aromatase inhibition. Ovariectomized mice performed worse than intact control mice in the novel object recognition test but, surprisingly, letrozole treatment rescued this deficit.

## Significance Statement

Aromatase inhibitors are widely used drugs used to suppress estrogen biosynthesis in adjuvant therapy for estrogen receptor-positive breast cancer. This also reduces estrogen levels in the brain, where it normally functions as a neuromodulator. While successful in reducing cancer recurrence rates, it is associated with cognitive side effects, but it is unclear if there is a causal relation. We developed a mouse model of letrozole (an aromatase inhibitor) adjuvant therapy and show that the mice have slower synaptic turnover and reduced synaptic plasticity but performed as well as regular control mice in an object recognition test. This highlights the complex role of brain-derived estrogen, in that it affects some but not all aspects of neuronal functioning.

## Introduction

17β-estradiol (E2) is the main female sex hormone produced in ovarian follicles. However, E2 is also produced in other tissues, including the brain (Hojo et al., 2004, 2008). Serum E2 levels largely reflect follicular E2 production and fluctuate with the estrous/menstrual cycle. In contrast, brain-derived E2 is regulated by neuronal and network activity (Hojo et al., 2004; Brann et al., 2011; Lu et al., 2019). All endogenous estrogens are synthesized from androgens (androstenedione or testosterone) by the enzyme aromatase (Cyp19A). Chronic aromatase inhibition, for example, with the drug letrozole, is used clinically in adjuvant therapy for estrogen receptor-positive breast cancer [in premenopausal women, aromatase inhibition must be combined with ovarian suppression; Early Breast Cancer Trialists’ Collaborative Group (EBCTCG), 2022]. E2 signaling can support tumor growth and thus cancer recurrence (Haynes et al., 2003; Spring et al., 2016; Korde et al., 2021), and it can directly initiate tumor formation (Lee et al., 2023). Women typically take aromatase inhibitors for many years, and there are reports of adverse effects on cognition and mood associated with its use (Weis et al., 2009, Phillips et al., 2010). However, it is often difficult to disentangle these symptoms from other effects of cancer and cancer treatment (surgeries, cytotoxic chemotherapy, antibody therapy, radiation). Here we have sought to develop a mouse model for chronic aromatase inhibitor treatment to examine the effects of aromatase inhibition in isolation, without the confounders inherent in clinical studies.

Ovariectomy—the surgical removal of the ovaries—and the associated large drop in circulating E2 lead to a loss of dendritic spines in pyramidal cells of hippocampal area CA1 and motor, somatosensory, and prefrontal cortex of rodents (but not occipital cortex, area CA3, and hippocampal granule cells), and this spine loss can be reversed by exogenous E2 (Gould et al., 1990; Chen et al., 2009; Ye et al., 2019). Spine density also fluctuates during the estrous cycle in CA1 pyramidal cells, but not in pyramidal cells of the neocortex (Woolley et al., 1990; Markham and Juraska, 2002; Ye et al., 2019).

E2 affects neuronal function in at least two different ways. First, it generally promotes neuronal excitation through both pre- and postsynaptic mechanisms (Smejkalova and Woolley, 2010; Snyder et al., 2011; Oberlander and Woolley, 2016; Ye et al., 2019), and it increases the magnitude of hippocampal LTP (Warren et al., 1995; Córdoba Montoya and Carrer, 1997). This augmented glutamatergic signaling can promote spine formation (Schwarz et al., 2008) and also decreases seizure threshold in experimental animals and humans under certain conditions (Herzog et al., 1997; Scharfman and MacLusky, 2006; Velíšková and DeSantis, 2013; Sato and Woolley, 2016). Secondly, E2 is anti-inflammatory, with neuroprotective effects in a wide range of disease models including traumatic brain injury and Alzheimer’s disease (Fillit et al., 1986; Azcoitia et al., 2019; Duncan , 2020; Kövesdi et al., 2020). Consequently, E2 affects cognition by supporting learning and memory and slowing cognitive decline (Luine, 2014; Broestl et al., 2018; Itoh et al., 2023).

Ovariectomized rodents are used to study the role of brain-derived E2 without the “background” of circulating follicular E2. To further control E2 levels, the biosynthetic enzyme aromatase can be targeted genetically (Lu et al., 2019) or pharmacologically. Aromatase inhibition suppresses E2 synthesis in the brain and adipose tissue (Bhatnagar, 2007) and recapitulates some effects of ovariectomy, such as reduced seizure threshold, LTP deficits, reduced spine density, and neuroinflammation (Vierk et al., 2012, Sato and Woolley, 2016; Lu et al., 2019, Lu et al., 2020). The effect of aromatase inhibition on behavior and cognition in rodents are variable and include impairments, no change, or improvements (reviewed in Rosenfeld et al., 2018). Here, in order to better understand the role of brain-derived E2 in brain plasticity in general and on dendritic spine dynamics in particular, we have used ovariectomized mice in which we also inhibited E2 biosynthesis using the aromatase inhibitor letrozole together with dynamic measurements of neocortical dendritic spines, LTP, and performance in the novel object recognition test.

## Materials and Methods

### Animals

All animal procedures were approved by our Institutional Animal Care and Use Committee. Thy1-EGFP-M mice (Jackson Laboratory #007788; RRID:IMSR_JAX:007788) were group housed (up to five per cage) on a 12 h light/dark cycle with access to food and water *ad libitum*. To inhibit the E2-synthesizing enzyme aromatase, mice were treated with letrozole (Tocris) at a concentration of 1 mg/kg via daily intraperitoneal injection, estimated to result in plasma concentrations in the range of ∼500 ng/ml (Taheri et al., 2024). Letrozole was dissolved in DMSO at a concentration of 3 mg/ml and diluted 1:10 in saline prior to injection. Vehicle injections consisted of 1:10 DMSO/saline only. There is currently no “gold standard” letrozole dose for rodents, but generally, researchers use dosages in the range of 0.1–10 mg/kg (Zhou et al., 2010; Vierk et al., 2012; Mamczarz et al., 2024). Experiments were performed in adult mice, and all ovariectomies were performed in mice older than 10 weeks.

### Serum/hormone assays

Mice were anesthetized with ketamine/xylazine (100 and 10 mg/kg, respectively). For serum preparation, between 1 and 1.5 ml of blood was collected from the left ventricle using a 2 ml syringe. Blood was left undisturbed to coagulate for 45 min at room temperature and subsequently centrifuged at 1,000 × *g* for 12 min at 4°C. The supernatant (serum) was removed and sent to the University of Virginia Ligand Core Laboratory for analysis using ELISA assays.

### Ovariectomy

Ovariectomies were performed in mice 10–16 weeks old under isoflurane anesthesia (1–2%, delivered via nose cone). Animals received a subcutaneous injection of dexamethasone (0.1 mg in 0.05 ml solution). At this age they are considered adult (Bell, 2018). Animals were placed on their back on a 32°C heating pad, and their abdomen was shaved and disinfected with iodine solution. A vertical ∼1 cm incision was made in the lower abdomen along the midline, the skin was gently separated from the underlying muscle using blunt forceps, and lidocaine (0.5 ml, 2% w/v) was injected between the skin and muscle layers. Then, an incision of similar size and the same orientation was made through the abdominal muscles and peritoneum, and both ovaries were isolated and removed. Both incisions (muscle and skin) were then sutured separately using resorbable PGA sutures (Dynarex). Following surgery, mice received subcutaneous injections of Baytril (2.5 mg/kg) to prevent infections and buprenorphine (0.3 mg/ml) to alleviate pain.

### Cranial window surgery

To prepare cranial windows, 3–4-month-old female Thy1-GFP-M mice were anesthetized with 1–2% isoflurane and placed on a 32°C heating pad in a stereotaxic device (Stoelting) where isoflurane was continually administered via a nose cone. A subcutaneous injection of dexamethasone (0.1 mg in 0.05 ml solution) was administered prior to surgery to reduce swelling, and lidocaine (0.5 ml, 2% w/v) was injected under the scalp to alleviate pain. The head was shaved and disinfected with iodine solution, and the skull was exposed by removing a ∼1 cm diameter region of scalp centered on the right hemisphere. A 2.1 by 2.1 mm region of bone over the right hemisphere (anteromedial corner at ML 0.5; AP 0.5) was removed by cutting the bone slowly using a #11 surgical blade while repeatedly bathing the skull in saline. After removal of the bone fragment, Surgifoam (Johnson & Johnson) soaked in saline was used to stop bleeding from the surrounding tissue. A square 2 mm^2^ glass coverslip was placed inside the craniotomy and glued into place using Vetbond tissue adhesive. A metal headmount was then glued onto the skull using dental cement. Following surgery, mice received subcutaneous injections of Baytril (2.5 mg/kg) to prevent infections and buprenorphine (0.3 mg/ml) to alleviate pain. Two-photon microscopy was started no sooner than 3 weeks after the surgery to allow the surgical inflammation to resolve.

### Two-photon microscopy

Dendritic spines of EGFP-expressing pyramidal neurons were imaged using a two-photon microscope (Sutter Instrument) equipped with a Mai Tai DeepSee Laser (Spectra-Physics) tuned to 920 nm and a 20×, 1.0 NA water-dipping objective (Zeiss). The microscope was controlled using ScanImage software v3.8 running in Matlab (MathWorks). Mice, under anesthesia with 1.0–1.2% isoflurane–oxygen mixture, were placed on a 32°C heating pad and head-fixed on the microscope stage using their headmounts.

*Z*-stacks were acquired starting from the pial surface and continuing to a depth of ∼100 µm with a step size of 0.75 µm. All images were acquired at a resolution of 1,024 × 1,024 pixels (0.108 µm/pixel; dwell time 1.6 µs). The same imaging volumes of interest were identified across many days as guided by imaging the brain surface vasculature with a CCD camera.

Two-photon images were analyzed using MapManager software (http://mapmanager.net; Cudmore et al., 2017) written in Igor Pro (WaveMetrics). Dendritic segments were identified and registered in the raw image stacks using a modified version of the Simple Neurite Tracer Plugin in FIJI. Spines were manually marked as a 3D point at their tip and semiautomatically connected to the dendritic backbone. This connection point gives each spine a distance (in μm) along the dendritic backbone from a manually identified fiduciary point common to all time points and was used to semiautomatically identify corresponding spines from one image stack to the next. Finally, the correspondence of spines between time points was visually verified and manually edited for errors. The rate of spines added and lost was calculated based on the number of spines that appeared or disappeared, divided by the total number of spines present. The turnover rate was calculated as the average of the spine loss rate and the spine addition rate. Only spines protruding by at least 0.5 µm (5 pixels) from the dendritic backbone were included in the final analysis.

### Immunohistochemistry and fixed tissue imaging

For fixed tissue quantification of spine density, female ovariectomized and letrozole- or vehicle-treated Thy1-EGFP-M mice were anesthetized with ketamine/xylazine (see above) and perfused transcardially with PBS followed by 4% phosphate-buffered paraformaldehyde (PFA). Brains were removed, postfixed in 4% paraformaldehyde overnight at 4°C, and equilibrated in 30% sucrose in PBS for >48 h. Brains were sectioned at 40 μm thickness on a sliding microtome (Leica), mounted on Superfrost Plus microscope slides (Fisher), coverslipped using ProLong Antifade Diamond mounting media (Invitrogen) and stored at 4°C. Confocal *z*-stack images were acquired on a Zeiss LSM 800 microscope and dendrite segments were analyzed using MapManager software (Cudmore et al., 2017).

### Behavior

The novel object recognition test (Lueptow, 2017) was performed in a chamber (black plastic bin measuring 30 × 40 cm, with 25 cm high walls) placed in a quiet, dimly lit area. Mice were placed in the empty chamber for 10 min, 1 d prior to the start of the experiment for acclimation. On Day 1 of the test (baseline day), two identical objects (100 ml Pyrex bottles with orange lids marked L and R on the lid to distinguish them to the experimenters) were placed into the lower left and upper right quadrants of the chamber, and the mice were allowed to explore for 10 min. The objects were always in the same positions (R and L) and the chamber was always oriented in the same way within the room with distal visual cues present. Twenty-four hours after the previous session, mice were placed into the chamber with the old object marked “L” in its old position and a novel object (a tower of five Lego Duplo bricks) in the other position, again for 10 min (test day). Objects were chosen to have similar dimensions and surface complexity. A cohort of naive control mice was subjected to the 10 min acclimation session and then placed into the chamber 24 h later with the two different objects (as during the test day) to determine whether animals had an innate preference for one of the objects. Sessions were filmed for later analysis using a mobile phone placed above the chamber. Chamber and objects were thoroughly cleaned after each use to eliminate odor cues. For data analysis, we quantified the time animals spent exploring each object on the two test days. Exploring consisted of time spent with the head oriented toward to object at a distance of no more than 1 cm. We did not score time spent grooming (even if that occurred within 1 cm and facing the object) or time spent perched on top of an object.

### Electrophysiology

Brain slices were prepared using a modified *N*-methyl-d-glucamine (NMDG)-based protective recovery method (Ting et al., 2018). Mice were deeply anesthetized using 5% isoflurane, and brains were removed and then submerged in ice-cold NMDG-HEPES-aCSF containing the following (in mM): 92 NMDG, 2.5 KCl, 1.25 NaH_2_PO_4_, 30 NaHCO_2_, 20 HEPES, 25 glucose, 2 thiourea, 5 Na-ascorbate, 3 Na-pyruvate, 0.5 CaCl_2_, 10 MgSO_4_, titrated to pH 7.35 with HCl and bubbled with carbogen (95% O_2_/5% CO_2_). Coronal neocortical slices (350 µm thick) were sectioned on a VT 1200S Vibratome (Leica) at 4°C in ice-cold NMDG-HEPES-aCSF solution and transferred into a recovery chamber filled with 150 ml of NMDG-HEPES-aCSF warmed to 32°C and bubbled with 95% O_2_:5% CO_2_. After 10 min of recovery, we started to gradually add concentrated NaCl, totaling 2 ml of 2 M NaCl in NMDG-HEPES-aCSF over a 15 min period (Ting et al., 2018). Slices were then transferred into room temperature holding aCSF containing the following (in mM): 92 NaCl, 2.5 KCl, 1.25 NaH_2_PO_4_, 30 NaHCO_2_, 20 HEPES, 25 glucose, 2 thiourea, 5 Na-ascorbate, 3 Na-pyruvate, 2 CaCl_2_, 2 MgSO_4_, titrated to pH 7.35 with NaOH and bubbled with 95% O_2_:5% CO_2_. Slices were incubated at room temperature for at least 1 h before recording. For field potential recordings, slices were transferred into a submerged recording chamber and continually superfused at a rate of ∼2 ml/min with aCSF containing the following (in mM): 126 NaCl, 2.5 KCl, 1.25 NaH_2_PO_4_, 26 NaHCO_3_, 1 MgCl_2_, 2 CaCl_2_, and 10 glucose, equilibrated with 95% O_2_:5% CO_2_. All experiments were conducted at 32°C. Signals were acquired using an Axon MultiClamp 700B amplifier, Digidata 1440A digitizer, and pClamp software, sampled at 10 kHz, and filtered at 3 kHz. Extracellular field potentials were evoked using a bipolar concentric stimulating electrode (FHC) placed in layer 4 and recorded in cortical layer 2/3 using glass micropipettes (≈1 MΩ) filled with aCSF. Pipettes were pulled on a Sutter P-87 puller using borosilicate glass (1B150F-4, World Precision Instruments). Stimulation consisted of 500 µs constant voltage pulses delivered by a WPI stimulus isolator. Field potentials were evoked once every 20 s, and stimulation intensity was adjusted such that the control field potential amplitude was between 0.1 and 1 mV. Each slice was recorded for 20–30 min prior to starting the LTP measurement protocol, and slices with drifting or unstable baseline fEPSPs were rejected. Theta burst stimulation to induce LTP consisted of bursts of four pulses delivered at 100 Hz, trains of 10 bursts each were delivered at 5 Hz, and trains were repeated six times at 0.1 Hz, resulting in a total of 240 stimulation pulses. The following pharmacological reagents were applied via the bath solution: drugs (all Tocris) were bath applied—GABA(A) receptor antagonist picrotoxin (50 µm), AMPA/NMDA antagonist kynurenic acid (10 mM), sodium channel antagonist TTX (1 µm).

### Experimental design and statistical tests

Only female mice were used in this study (except for some of the control mice used in the novel object recognition test), as we investigated the main female sex hormone in a female context and used a model of breast cancer treatment, with >99% of breast cancers occurring in women (Miao et al., 2011). For imaging of dendritic spines, sample size refers to the number of animals, and we analyzed between 197 and 289 μm of secondary apical (layer 1) dendrite per animal, depending on the amount of clearly visible dendritic segments. For slice physiology, sample size reflects the number of slices, with one measurement per slice. Between one and five (median: 2.5) slices per animal were included in the analysis, with six animals each per group. For behavioral experiments, sample size refers to the number of animals. We did not assume that our data was normally distributed; thus, for statistical analysis comparing two experimental groups, we used Mann–Whitney *U* tests for two unpaired datasets, Wilcoxon signed rank tests for two paired datasets, and Kruskal–Wallis test with Dunn’s post hoc analysis for comparison of more than two datasets.

## Results

Surgical ovariectomy reduces the density of dendritic spines in neurons of the mammalian neocortex and hippocampus, and exogenous E2 can reverse this effect (Gould et al., 1990; Chen et al., 2009; Ye et al., 2019). Although ovarian follicles are the major source of E2 in females of reproductive age, steroid hormones are produced in other tissues as well. Notably, E2 is synthesized in neurons and glia, and this brain-derived E2 functions as a neuromodulator (Taxier et al., 2020).

### Letrozole treatment reduces E2 serum levels

**Figure 1.**
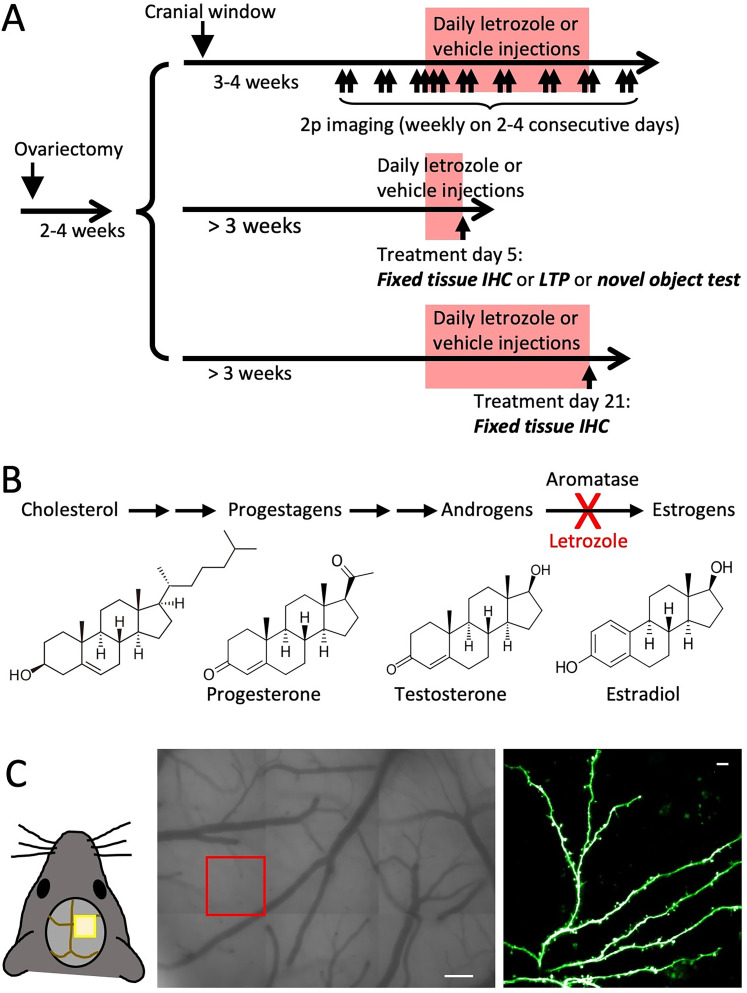
Experimental design. ***A***, Timeline indicating time of ovariectomy, cranial window implantation, letrozole/vehicle treatment (pink box), and two-photon imaging sessions (arrowheads). ***B***, Schematic depicting steroid hormone biosynthesis. Letrozole inhibits the conversion of androgens to estrogens via aromatase (Cyp19). ***C***, Left, Mouse with an implanted cranial window. Center, Tiled light microscopic images showing surface vasculature used as a “roadmap” to find the same location for two-photon imaging on successive imaging sessions. Scale bar = 50 μm. Right, Example two-photon *z*-stack maximum intensity projection showing sparsely labeled GFP-positive dendrites, scale bar = 5 μm.

**Figure 2.**
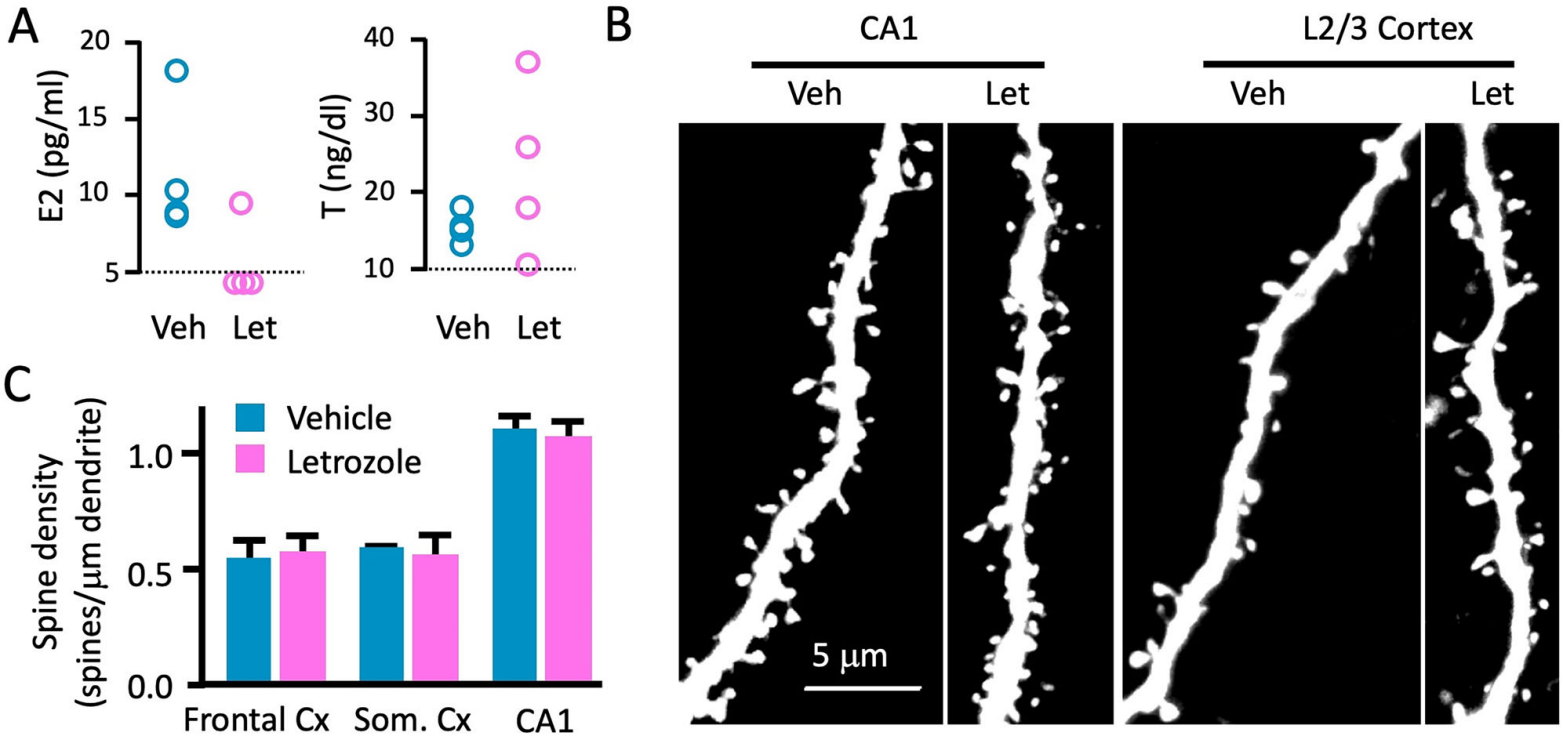
Letrozole treatment alters plasma neurosteroid levels but does not affect spine density in fixed tissue. ***A***, E2 and testosterone serum levels determined by ELISA in vehicle- and letrozole-treated mice (*n* = 4 each). The assay detection limit is marked by the dashed lines. ***B***, Representative confocal images from fixed tissue sections showing apical dendrites of eGFP-expressing layer 5 pyramidal cells in Thy1-GFP-M mice treated with letrozole and vehicle for 21 d. Images were acquired in L2/3 somatosensory cortex and the CA1 region of the hippocampus. Note the higher spine density in hippocampal neurons. ***C***, Summary of spine density counts from fixed tissue sections in frontal cortex, somatosensory cortex, and CA1 in letrozole- and vehicle-treated mice. Similar proximo-distal dendritic segments were analyzed in the two groups. There were no significant differences in any region in either treatment group (*n* = 6 animals/group; frontal cortex, *p* = 0.837; somatosensory cortex, *p* = 0.476; CA1, *p* = 0.931; Mann–Whitney *U* test).

Here, we have investigated the effects of pharmacological suppression of E2 synthesis in ovariectomized 4–5-month-old female mice. One month after ovariectomy, we began treatment with daily intraperitoneal injections of letrozole (30 μl total; 1 mg/kg) or vehicle (10% DMSO in saline). For an experimental timeline, see Figure 1*A*. Letrozole is a competitive inhibitor of aromatase, the enzyme that catalyzes the conversion of testosterone to E2 (Fig. 1*B*). As letrozole readily crosses the blood–brain barrier, intraperitoneal letrozole can efficiently target aromatase within the CNS (Dave et al., 2013; Arora et al., 2019). In our mice, serum E2 levels were significantly reduced after 1 week of daily letrozole treatment, with most mice having levels below the ELISA assay’s detection limit of 5 pg/ml (controls: 11.5 ± 3.2 pg/ml, range, 8.7–18.1; letrozole: <6.125 pg/ml, range, <5–9.5; *n* = 4 each; Fig. 2*A*). Testosterone, the substrate for aromatase, was also quantified, to ascertain whether aromatase inhibition might lead to an accumulation of testosterone. While vehicle controls all had very similar testosterone levels (15.7 ± 1.5 ng/dl; *n* = 4), those of letrozole-treated mice exhibited greater variation (23.0 ± 8.0 ng/dl; *n* = 4; Fig. 2*A*) that was compatible with both, an increase, and unchanged levels compared with control.

### Letrozole treatment reduces dendritic spine turnover but does not affect spine density

Initially, we quantified spine density in somatosensory cortex, frontal cortex, and the CA1 region of the hippocampus using fixed tissue sections from ovariectomized Thy1-GFP-M mice that had been treated with letrozole or vehicle for 3 weeks (Fig. 2*B*,*C*). We chose tertiary or higher order dendritic segments within the apical tuft of layer 5 pyramidal cells and within the distal tuft of CA1 pyramidal cells in dorsal hippocampus (AP −1.5 to −2), totaling, on average, 324 μm of length per region per animal (range, 150–529 μm; Fig. 2*C*; *n* = 3 mice/group) and quantified spine density. Since ovariectomy leads to a reduction in spine density (Ye et al., 2019), we wanted to test whether further lowering E2 levels via aromatase inhibition would exacerbate this effect. However, spine density in all three regions did not differ between letrozole and vehicle treatment groups (spine densities, in count/micrometer vehicle vs letrozole treatment; frontal cortex, 0.55 ± 0.08 vs 0.57 ± 0.07, respectively; somatosensory cortex, 0.59 ± 0.01 vs 0.56 ± 0.09; CA1, 1.10 ± 0.06 vs 1.07 ± 0.07).

While fixed tissue sections allow the analysis of multiple anatomical areas, they do not permit longitudinal studies of spine density and investigations of spine dynamics. Therefore, to follow up on our initial findings, we implanted cranial windows above the somatosensory cortex of ovariectomized Thy1-GFP-M mice to observe spines on the apical tufts of layer 5 pyramidal cells before, during, and after aromatase inhibition or vehicle treatment using in vivo two-photon microscopy (Fig. 1*C*).

**Figure 3.**
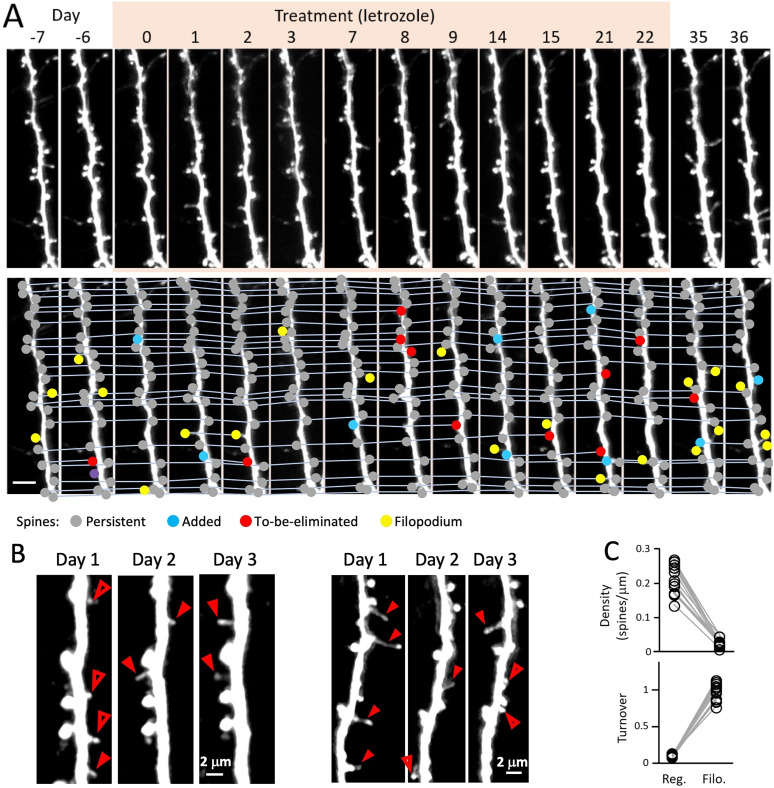
In vivo longitudinal imaging of spines on apical dendritic tufts. ***A***, Top panel, A series of two-photon images showing the same apical dendritic tuft segment of a layer 5 pyramidal neuron in the primary somatosensory cortex of a Thy1-GFP-M mouse over the course of 43 d. Images were acquired during the baseline period, letrozole treatment (indicated by tan box), and recovery after treatment. Scale bar, 5 μm. Bottom panel, These are the same images as above, but with spines color coded for analysis. Gray dots mark persistent spines (those also observed in the previous and the following image). Blue dots denote new spines that were not seen in the previous image. Red dots indicate spines that will be eliminated in the following image. Yellow dots denote filopodia-like spines. ***B***, Filopodia-like spines have different dynamics compared with “regular” spines. Example images of two dendritic segments from two animals acquired on 3 consecutive days each. Filopodia (long thin spines) are marked with red arrowheads and are all transient, i.e., only seen in one image. Open red arrowheads in the left image indicate regular spines that were eliminated on the following day. ***C***, Top panel, Quantification of the density of regular spines and filopodia, showing a ∼ 10-fold higher density of regular spines. Summary of all animals imaged during the baseline period [before letrozole (*n* = 8) or vehicle (*n* = 6) treatment]. Bottom panel, Turnover rate for regular spines (*n* = 932 spines from 14 mice) and filopodia (*n* = 156 filopodia from 14 mice) from data shown in the left panel (turnover, probability of addition or elimination per spine per day). Filopodia have a >10-fold higher turnover rate. These measurements indicate that despite their much lower density, filopodia contribute >50% of the overall turnover rate for all spines.

The top panel in Figure 3*A* shows a representative spine segment in a letrozole-treated mouse imaged 15 times over a period of 6 weeks with images acquired on at least 2 consecutive days each week. The same images are shown again in the bottom panel of Figure 3*A* but overlayed with annotation classifying each spine (persistent spine, added or to-be-eliminated spine, filopodia-lie spine, see below) and lines connecting identical spines in each image across time. All acquired images were used to determine spine density, whereas spine dynamics (i.e., addition, subtraction, and turnover) were only calculated from images acquired on consecutive days, as unequal imaging intervals will distort measured turnover rates due to different proportions of missed events.

Before beginning our analyses of the effects or aromatase inhibition, we had to account for the fact that spines fall into two categories with drastically different turnover rates: regular spines (comprising short, stubby, and mushroom types), with low turnover probabilities of <0.1/day, and filopodia-like long thin spines with a high turnover rate of nearly 100%/day. Filopodia-like spines are thought to be preliminary versions of spines that may not form synapses and only sometimes become established as regular, synaptic terminal-bearing spines (Ziv and Smith, 1996; Portera-Cailliau et al., 2003; reviewed in Berry and Nedivi, 2017). Although regular spines outnumber filopodia-like spines by a factor of ∼10, their lower turnover rates mean that over 50% of total turnover events derive from filopodia-like spines (Fig. 3*B*,*C*). Therefore, we analyzed spine density and dynamics separately for regular spines and filopodia-like spines.

**Figure 4.**
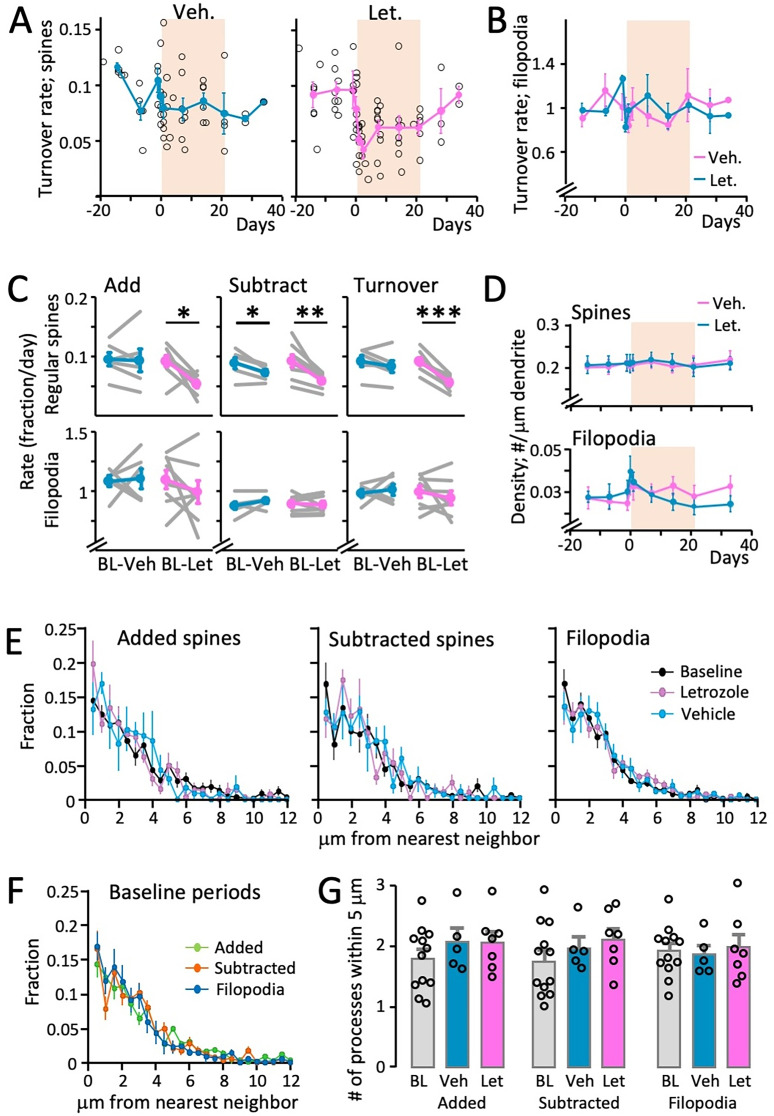
Letrozole treatment reduces spine turnover. ***A***, Left panel, Spine turnover in vehicle-treated mice (regular spines only). Black circles represent all individual spine turnover measurements in six mice over the course over up to 56 d encompassing 21 d of vehicle treatment (tan box). Please note that not all animals were followed the entire time and that measurements did not all occur on the exact same days in all animals. Teal line represents binned averaged turnover rates (2–4 d bins) and shows no effect of vehicle treatment. Right panel, Same as in the left panel, but for letrozole-treated mice (*n* = 8). The pink line represents the binned averaged data. Spine turnover is reduced during letrozole treatment (*p* < 0.05 for letrozole treatment on Days 1, 2, and 14, compared with baseline period; Kruskal–Wallis test) and recovers after treatment cessation. Error bars are SEM. ***B***, Turnover rates of filopodia are not affected by vehicle (teal) or letrozole (pink). The tan box indicates the treatment period. Data points are binned (2–4 d bins) averages per animal (*n* = 6 for vehicle and 8 for letrozole treatment); error bars are SEM. There were no significant differences during treatment or between treatment groups; Kruskal–Wallis test. ***C***, Top, Rates of spine addition (fraction of new spines/day), subtraction (fraction of spines to-be-deleted/day), and turnover (average of addition + subtraction) for each letrozole- and vehicle-treated animal before and during treatment (teal and pink: averages and SEM for vehicle and letrozole treatment, respectively; gray lines indicate data from each individual animal). Letrozole treatment decreased all three parameters, while vehicle-treated animals had a smaller but significant reduction in spine subtraction (significance levels of *0.05, **0.01, ***0.005; Mann–Whitney *U* test) Bottom, Addition, subtraction, and turnover of filopodia for each letrozole- and vehicle-treated animal before and during treatment. No significant changes were observed for any parameter in either group. ***D,*** Spine density is not affected by vehicle or letrozole treatment. Density of regular spines (top panel) and filopodia (bottom panel) in vehicle- (teal, *n* = 6) or letrozole-treated (pink, *n* = 8) animals. The tan box indicates the treatment period. Data points are binned (2–4 d bins) averages per; error bars are SEM. There were no significant differences during treatment or between treatment groups; Kruskal–Wallis test. Filopodia appeared transiently increased at the beginning of either treatment, but this did not reach statistical significance. Note the much higher density of regular spines compared with filopodia. ***E***, Letrozole or vehicle treatment does not affect location of spine turnover. Distance of each added spine (left panel), to-be-subtracted spine (middle panel), and filopodium (right panel) to its nearest neighbor during baseline imaging period (black), during letrozole (pink), and vehicle (teal) treatment, depicted as probability densities. Data shows averages, and error bars are SEM. There were no significant differences between baseline, letrozole, and vehicle periods for all three measurements. ***F****,* This panel depicts the baseline periods from the three measurements in panel ***E***, showing that added and subtracted spines, as well as filopodia, are similarly distributed with regard to their distance to the next closest spine. This means that neither occurs preferentially close to, or preferentially far away, from existing spines. ***G,*** Letrozole or vehicle treatment does not affect spine clustering. The number of processes (spines or filopodia) within 5 μm for each added (left) and subtracted (center) spine and each filopodium (right) during the baseline imaging period (gray) and during vehicle (teal) and letrozole (pink) treatment. Circles depict data from each animal, and bars show averages/SEM. There were no significant differences between treatments and between addition/subtraction/filopodium.

Quantification of spine density from eight letrozole- and six vehicle-treated mice (average of 289 ± 12 μm dendrite/animal) over a period of 6–7 weeks before, during, and after treatment confirmed the results obtained in fixed tissue analysis: spine density remained stable and was not affected by aromatase inhibition. This was seen both for regular and filopodia-like spines (Fig. 4*D*). However, we found that turnover rates were transiently reduced throughout letrozole, but not vehicle treatment, and recovered after treatment cessation (Fig. 4*A*; spine turnover during letrozole treatment differs significantly from baseline on treatment days 1, 2, and 14, *p* < 0.05, Kruskal–Wallis test). This effect was restricted to regular spines; the high turnover rates of filopodia-like spines were unaffected (Fig. 4*B*). As turnover rates are calculated by averaging addition and subtraction rates, we analyzed these parameters separately (Fig. 4*C*, letrozole or vehicle). Letrozole treatment resulted in a roughly equal reduction of both addition and subtraction of regular spines (down 42%, *p* < 0.05 and 37%, *p* < 0.01, respectively), while vehicle treatment did not affect addition but also slightly reduced spine subtraction (−18%, *p* < 0.05; all comparisons here using Mann–Whitney *U* test). Addition and subtraction of filopodia was unaffected (Fig. 4*C*, bottom panel).

To further characterize spine dynamics, we determined whether or not added or subtracted spines and filopodia might be preferentially located near other spines. To this end, we extracted the distances between all spines and filopodia along each dendrite from our dataset during all baseline, letrozole, and vehicle imaging sessions. From this, we determined the nearest neighbor of each added spine, to-be-subtracted spine, and filopodium and calculated the fraction of nearest neighbors in 0.5 μm bins (Fig. 4*E*) under each condition. We found no differences in the probability densities of spines added or subtracted during baseline, vehicle, and letrozole treatment periods (Fig. 4*E*), as well as no significant differences between addition, subtraction, and filopodia during the baseline period (Fig. 4*F*; *p* > 0.05; Kruskal–Wallis test for all comparisons of treatments and spine type). We also quantified the number of spines within 5 μm of each added or subtracted spine and each filopodium (Fig. 4*G*) during the three treatment periods. Again, we found no significant differences between treatments or between addition/subtraction/filopodium. This indicated that letrozole treatment does not affect spine localization and clustering and also suggests that spine addition and subtraction in our dataset occurred with the same preference regarding proximity to other spines.

In summary, letrozole treatment reduces spine turnover (addition and subtraction), while leaving spine density and relative location unaffected.

### Letrozole treatment reduces neocortical LTP amplitude

As our results show that aromatase inhibition attenuates dendritic structural plasticity, we wanted to test whether it might impact synaptic and network function. Neurons and neuronal networks dynamically respond to changing inputs and can persistently alter their intrinsic and synaptic properties; this plasticity is hypothesized to be the basis of learning and memory. Experimentally, we can observe structural plasticity as changes in spine dynamics, density, and morphology and functional plasticity as changes in synaptic strength and intrinsic neuronal excitability. Since aromatase inhibition appears to impair structural plasticity, we wanted to explore whether functional plasticity within the same neuronal networks was affected as well. To this end, we used acute brain slices from letrozole- or vehicle-treated mice to measure long-term potentiation of layer 4 to layer 3 connections in somatosensory cortex using theta burst stimulation (Bear and Kirkwood, 1993; Kirkwood et al., 1993). Mice had been treated for 5 d, the time point at which robust reductions in spine dynamics were detectable.

**Figure 5.**
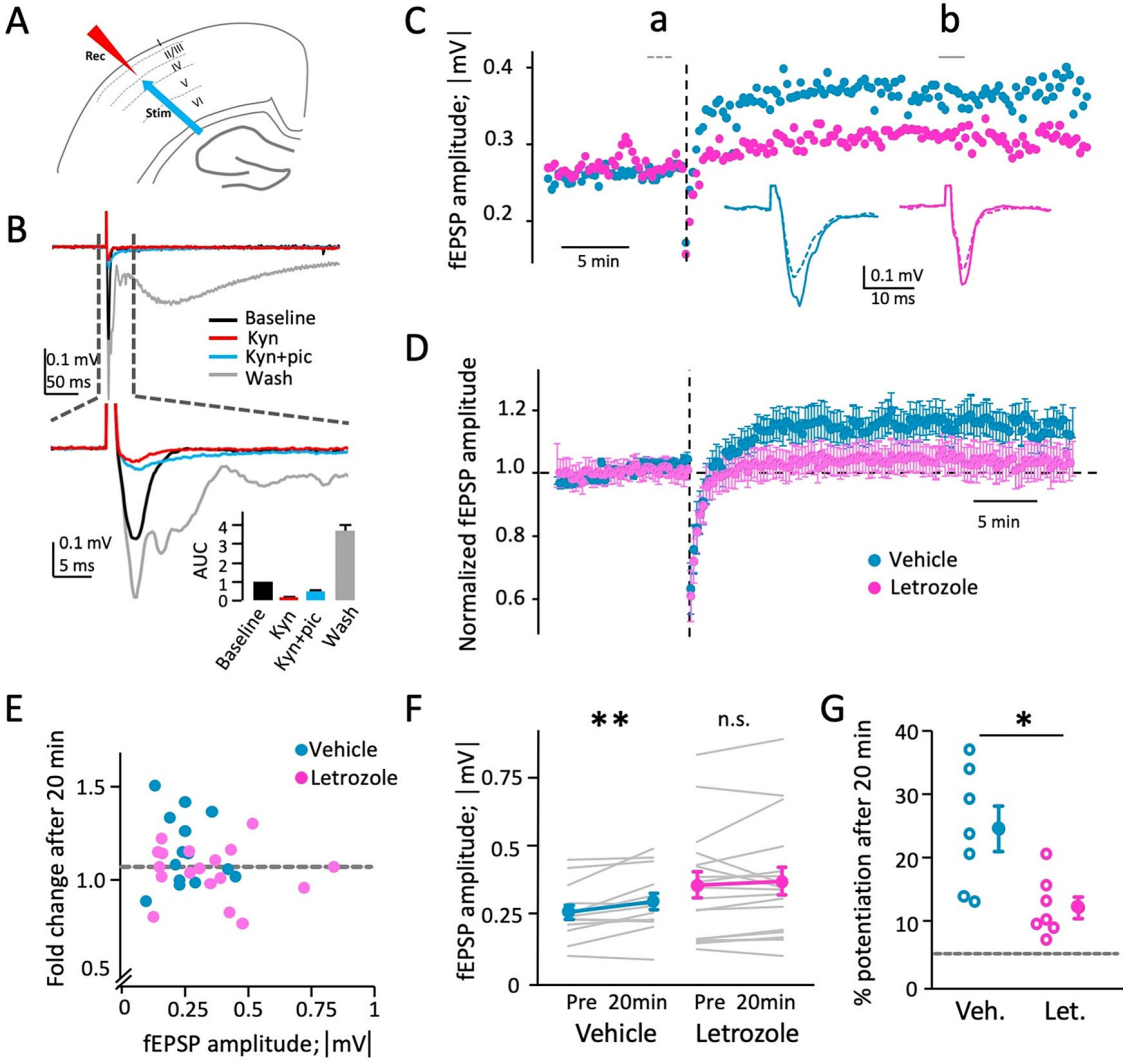
Letrozole treatment attenuates neocortical LTP. ***A***, Schematic depiction of stimulation (stim) and recording (rec) sites within layer 4 and layer 3 of somatosensory cortex (top), theta burst stimulation consisted of bursts of 4 pulses at 100 Hz, trains of 10 such bursts were delivered at with 200 ms interburst intervals, and six trains were delivered with 10 s intertrain intervals. ***B***, Pharmacology of fEPSPs (top), and a briefer period around stimulus surrounding the stimulation (bottom), and bar graph summarizing the area under the curve (AUC) of responses measured from 1 to 10 ms after the stimulus. The response is almost entirely eliminated by kynurenic acid (10 mM), indicating it is mostly glutamatergic, slightly disinhibited by picrotoxin (50 μM) in the presence of kynurenic acid, and severely disinhibited when kynurenic acid is washed out, transforming the response into a large, polyphasic waveform (picrotoxin does not readily wash out). ***C***, LTP induction in representative slices from a vehicle (teal) and letrozole-treated (pink) animal. The time of theta burst stimulation is indicated by the dotted vertical line. The fEPSP amplitude increases after theta burst stimulation. Example traces before (dotted line) and after (solid lines) theta burst stimulation indicated as time points a and b, show increased fEPSP amplitude after LTP induction for both slices, but stronger potentiation in the slice from the vehicle-treated animal. ***D***, Average fEPSP amplitudes in all slices from vehicle (teal) and letrozole (pink) treated animals, normalized to baseline. Theta burst stimulation is indicated by dotted vertical line. Error bars = SEM. Letrozole-treated slices show reduced/absent LTP. ***E***, LTP magnitude does not depend on fEPSP amplitude. Scatterplot depicting absolute values of fEPSP baseline amplitude (average of last 10 responses immediately before theta burst stimulation) versus fold change after theta burst stimulation (average of 10 responses 20 min after theta burst stimulation) in all slices from vehicle-treated (teal, *n* = 14 slices from 7 animals) and letrozole-treated (pink, *n* = 18 slices from 7 animals) animals. Five percent potentiation threshold for successful LTP induction is indicated by a dashed gray line. There is no robust correlation between LTP magnitude and initial fEPSP amplitude (*R*^2 ^= 0.03 for letrozole treatment; *R*^2 ^= 0.02 for vehicle treatment). ***F***, fEPSC amplitudes in all slices from vehicle- and letrozole-treated animals, before and after LTP induction protocol (average of 10 responses each immediately before, and 20 min after theta burst stimulation; see panel ***E***). Individual slices are shown as gray lines, and averages are indicated in teal (vehicle treatment group) and pink (letrozole treatment group). Responses in the vehicle group were significantly potentiated, and those in the letrozole group were not (paired *t* test). ***G***, Plot summarizing fEPSC potentiation in responding slices from vehicle- (teal) and letrozole-treated (pink) animals (open circles) and average values (filled circles) ±SEM. LTP in letrozole-treated slices is significantly reduced. Five percent threshold for LTP induction is indicated with dashed gray line (*p* < 0.05; Mann–Whitney *U* test).

Electrical stimulation in layer 4 evoked brief field PSPs (fPSPs) in layer 3 (see Fig. 5*A* for a schematic). We bath applied a series of synaptic blockers to characterize fPSPs pharmacologically (Fig. 5*B*). The general ionotropic glutamatergic receptor antagonist kynurenic acid (10 mM) blocked ∼90% of the fPSP, showing that it was indeed glutamatergic. Addition of the GABA_A_ receptor antagonist picrotoxin (50 μM) resulted in a small increase of fPSP amplitude and a significant increase in decay kinetics, due to disinhibition. While kynurenic acid readily washes out of slice preparations, picrotoxin does not. Thus, during washout, the residual fPSP transformed into a large, polyphasic, epileptiform discharge, typical of a disinhibited network. In summary, our stimulation evokes a brief, reproducible fEPSP shaped by direct activation of glutamate receptors and recruitment of GABAergic inhibition.

We recorded fEPSPs for 20–30 min prior to starting the LTP measurement protocol, and slices with drifting or unstable baseline fEPSCs were rejected. We obtained stable recordings in a total of 32 slices from 12 animals (6 letrozole, 6 vehicle treated for 5 d). In 14/32 slices (44%; vehicle, 7/14; letrozole, 7/18), theta burst stimulation in layer 4 resulted in long-term potentiation of fEPSPs in layer 3, defined as a long-term increase of >5% in fEPSC amplitude. In the remaining slices, theta burst stimulation had no effect or caused a reduction of fEPSP amplitude. Figure 5*C* shows example recordings from slices from vehicle- and letrozole-treated animals. Theta burst stimulation potentiated the responses in both slices, but more so in the one from the vehicle treatment group. Figure 5*D* shows summary data for all slices, with greater potentiation in the vehicle treatment group. When comparing baseline fEPSC amplitude to the amplitude 20 min post theta burst stimulation, slices from the vehicle-treated group showed significant potentiation, while slices from the letrozole treatment group did not (vehicle: 15.7 ± 5.1%, *n* = 14, *p* < 0.01; letrozole: 4.6 ± 3.4%, *n* = 18, *p* = 0.33; Fig. 5*F*). When only including slices with at least 5% potentiation in both groups, there was a significantly smaller fEPSP amplitude increase after letrozole treatment (vehicle: 24.8 ± 3.5%; letrozole: 12.6 ± 1.7%; *n* = 7 each; *p* < 0.05; Fig. 5*G*). To ensure that this effect was not an artifact of between-group differences in baseline fEPSP amplitude, we plotted baseline amplitude against fold potentiation for all slices (those with and without detectable LTP; Fig. 5*E*). Regression analysis revealed no significant correlation between potentiation and baseline amplitude (*R*^2 ^= 0.02 for vehicle, 0.03 for letrozole). Thus, letrozole treatment resulted in a significant reduction in LTP magnitude compared with control (Fig. 5*F*).

**Figure 6.**
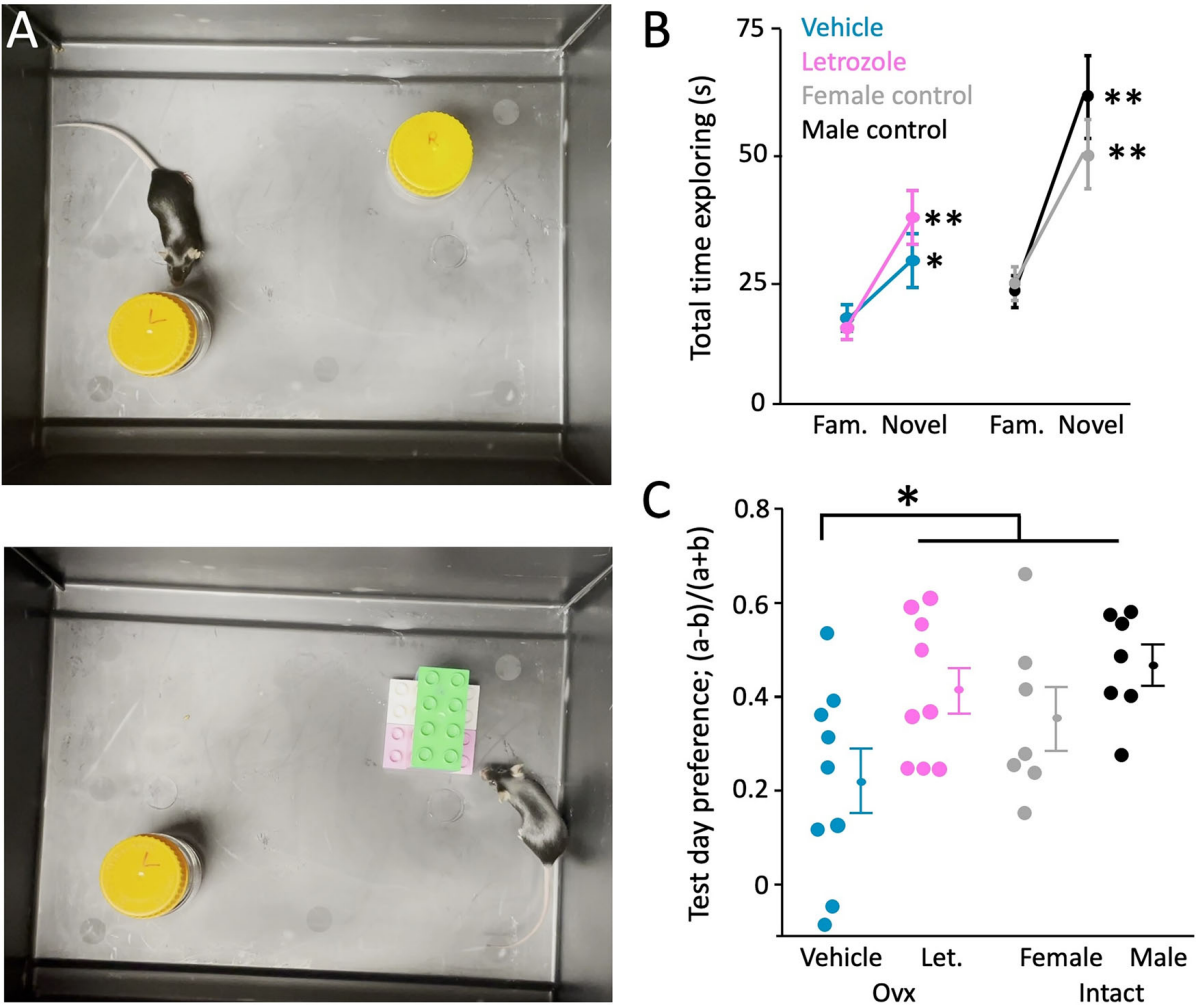
Letrozole treatment rescues the ovariectomy-induced deficit in the novel object recognition test. ***A***, Top, Experimental setup during control day. Two 100 ml Pyrex bottles were positioned in the lower left and upper right quadrant of the box, and caps were marked “L” and “R” so that the same bottle was always at the same location. Bottom, On test day (24 h later) the upper right bottle “R” was replaced by the novel object, a Lego tower, while the bottle marked “L” remained as the familiar object in its old location. Mice spent 10 min each with the objects on control and test day and time spent exploring each object was quantified. ***B***, Time spent exploring objects on test day. Vehicle- and letrozole-treated mice (left panel) and naive control mice (females in gray and males in black, right panel). All groups of animals showed a significant preference for the novel object (error bars = SEM; *p < 0.05; Kruskal–Wallis test). Male and female controls spent a greater total amount of time exploring than ovariectomized animals. ***C***, Preference index on test day (time_novel_ − time_familiar_ divided by time_total_) for each animal. A negative number indicates that the animal spent more time with the familiar object. Vehicle-treated mice had a significantly lower preference index (i.e., spent a smaller fraction of time exploring the novel object) than letrozole-treated animals. Letrozole-treated mice did not differ statistically from female and male controls.

### Letrozole treatment does not affect performance in the novel object recognition test

Aromatase inhibition leads to deficits in neocortical (this study) and hippocampal LTP (Vierk et al., 2012). We were curious to see whether this apparent widespread impact on plasticity would be accompanied by a behaviorally detectable deficit. To that end, we conducted a novel object recognition test on vehicle- and letrozole-treated ovariectomized mice and intact female and male controls. We placed animals into a chamber with two identical objects (two 50 ml glass bottles) for 10 min on Day 1 (baseline day) and with one of the familiar Day 1 objects and one novel object (Lego tower) for 10 min on Day 2 (test day, 24 h after control session). We quantified the amount of time animals spent exploring either object on both days. On average, animals spent the equal amounts of time with each identical object on control day (vehicle: 26.6 ± 3.6 s vs 24.0 ± 5.9 s, *n* = 9; letrozole: 28.3 ± 3.2 vs 31.8 ± 2.4 s, *n* = 9; female controls: 31.1 ± 4.0 vs 31.6 ± 4.0 s, *n* = 7; male controls: 29.2 ± 4.3 vs 31.2 ± 4.1 s, *n* = 7; left vs right bottle). On the test day, mice spent significantly more time with the novel object (Lego tower) compared with the familiar object (vehicle: 13.8 ± 2.1 vs 22.9 ± 4.3 s; letrozole: 12.2 ± 1.8 vs 29.8 ± 4.3 s; female control: 19.5 ± 2.8 vs 41.2 ± 5.8 s; male control: 18.2 ± 2.7 vs 50.8 ± 6.9 s; Fig. 6*B*, *p* < 0.05 or higher for all). The bias toward exploring the novel object is quantified as the preference index:

$$({\text{time}}_{\mathrm{novel}}-{\text{time}}_{\mathrm{familiar}})/({\text{time}}_{\mathrm{novel}}+{\text{time}}_{\mathrm{familar}}).$$

Glass bottles and Lego tower had roughly equivalent dimensions and surface complexity (Fig. 6*A*) and control mice exploring the two different objects for 10 min without a prior baseline day did not significantly prefer one object over the other [time exploring the bottle: 31.1 +/−2.8 s; Lego tower: 34.8 ± 2.0 s., *n* = 7; not significant (Wilcoxon and paired *t* test)]. The degree of preference for the novel object serves as a general measure of overall cognitive ability, learning, and memory (Lueptow, 2017). We initially expected that letrozole-treated mice would have a lower preference for the novel object. However, we found the opposite (Fig. 6*C*): letrozole-treated animals had a significantly higher preference for the novel object compared with the vehicle-treated ones (0.42 ± 0.05 vs 0.22 ± 0.07; *p* < 0.05; *n* = 9 each). In fact, their preference index was indistinguishable from that of naive control mice (females, 0.35 ± 0.07; males, 0.47 ± 0.04; *n* = 7 each).

## Discussion

In this study, we have examined the role of brain-derived E2 using a mouse model of ovariectomy and chronic letrozole treatment. Letrozole inhibits aromatase, the enzyme that converts androgens into estrogens, thus significantly reducing brain E2 levels (Kokras et al., 2018). Females with intact ovaries are unsuitable for these studies, as letrozole is unable to lower E2 levels sufficiently in the context of intact hypothalamic-pituitary-ovarian feedback (Guo et al., 2022; Silveira et al., 2022). Letrozole is widely used clinically in adjuvant breast cancer treatment. The standard of care for postmenopausal women with estrogen receptor (ER)-positive breast cancer is to receive antiestrogen adjuvant therapy for up to 10 years. The two most common classes of drugs for this are aromatase inhibitors, such as letrozole, and selective estrogen receptor modulators (SERMS) such as tamoxifen and raloxifene. Adjuvant therapy is highly effective in reducing cancer recurrence rates but is frequently accompanied by cognitive and affective side effects (Weis et al., 2009; Phillips et al., 2010). The neural basis for these side effects is poorly understood, and it is often difficult in the clinical setting to distinguish the effects of cancer and chemotherapy from those of adjuvant therapy (Brezden et al., 2000; Hedayati et al., 2012; Lange et al., 2019).

In addition to its role in reproduction as the major female sex hormone, E2 is a neuromodulator. It is synthesized by neurons and glia in the brain and is involved in many processes, including maintenance of dendritic spines, neuronal excitation, and protection against neuroinflammation. Aromatase expression and activity within the brain can be fine-tuned temporally and locally to meet specific demands; for example, in rodent hippocampus it is upregulated in response to glutamatergic activity (Hojo et al., 2004; Sato and Woolley, 2016). Gonadal steroid hormones, which are regulated as part of the hypothalamic-pituitary-gonadal axis, provide oscillating background levels of circulating hormones without regional specificity but still affect brain neurosteroid signaling. Circulating E2, for example, affects the density and activity of hippocampal E2 receptors, and its prolonged absence (due to ovariectomy or after menopause) can eventually lead to long-term deficiencies in receptor expression that cannot be rescued by exogenous E2 (Bean et al., 2015; Ma et al., 2020). Thus, E2 signaling in the brain is modulated by the interplay of gonadal and brain-derived sources. Aromatase expression in pyramidal cells and astrocytes—but not interneurons—in mammalian neocortex has been detected using RT-PCR (Stoffel-Wagner et al., 1999; Tabatadze et al., 2014) and immunohistochemistry (Yague et al., 2006; Lu et al., 2019), while other reports do not mention detection of aromatase activity in cortical areas (Wu et al., 2009).

To study the effects of E2 in the brain, a number of approaches have been taken. The main ones are as follows: (1) observation of normal neurophysiological changes during the estrous cycle—which is however confounded by varying levels of other steroid hormones, notably progesterone; (2) ovariectomy, i.e., the removal of the largest source of circulating sex hormones—again eliminating E2 along with progesterone; (3) targeted disruption of E2 biosynthesis via aromatase inhibition; and (4) pharmacological interventions using estrogen receptor modulators. There are key differences depending on the specific manipulation, as well as the brain region examined. For example, ovariectomy leads to a large reduction in dendritic spines on pyramidal cells in the hippocampal CA1 area and in most neocortical regions, but not in hippocampal area CA3 and the occipital cortex (Ye et al., 2019). Spine density fluctuates during the estrous cycle on CA1 pyramidal cells but not on neocortical neurons (Gould et al., 1990; Chen et al., 2009; Ye et al., 2019), even though both cell types respond to ovariectomy. Possibly the hormonal fluctuations during the estrous cycle are not steep or sustained enough to affect spine maintenance on neocortical neurons, there are ceiling and floor effects with regard to the amount of available E2, or fluctuations in the levels of other steroid hormones compensate for the lack of E2. Ovariectomized mice with a forebrain-specific aromatase knock-out mutation have reduced spine density in CA1 and neocortex (Lu et al., 2019), but we show here that spine density in these areas does not change in response to pharmacological aromatase inhibition. This may be attributable to a lack of E2 during development in the knock-out versus its acute effects in adults in our study. Ovariectomy and aromatase inhibition should be regarded as fundamentally different manipulations: ovariectomy removes the major source of circulating sex hormones in females and thus leads to a drop in the absolute amount of all circulating steroid hormones: aside from E2, the ovaries are also the major source of progesterone, whose metabolite allopregnanolone is a well-studied allosteric activator of GABA_A_ receptors (Reddy, 2010). Aromatase inhibition, on the other hand, targets E2 specifically, affects all E2 synthesis including brain-derived E2, and likely does not lead to a drop in other steroid hormones.

We initially hypothesized that, since ovariectomy leads to spine loss in neocortical pyramidal cells, additional reduction of brain-derived E2 via aromatase inhibition would lead to an even greater decrease in spine density. However, our results show that treating ovariectomized mice with letrozole over a period of 30 d did not decrease spine density (Fig. 2). This echoes the unchanged spine density on neocortical neurons during the estrous cycle (Ye et al., 2019). A potential explanation for this finding may be that there is a floor effect regarding the amount of E2—i.e., the residual amounts left after ovariectomy are insufficient to contribute to spine maintenance. Alternatively, letrozole treatment may shift the balance of neurosteroids from estrogens to its metabolic precursors, i.e., androgens and progestins. Notably, not only E2, but also progesterone and testosterone supplementation will rescue spine loss after ovariectomy (Luine et al., 2022). Although, to our knowledge, measurements of brain androgen and progesterone levels after letrozole treatment have not been reported, letrozole does increase progesterone and androgen levels in plasma and follicular fluid of patients undergoing adjuvant endocrine therapy (Rossi et al., 2009) or assisted reproductive procedures (Dallagiovanna et al., 2022). We hypothesize that in addition to a reduction in E2, aromatase inhibition may increase testosterone and/or progesterone levels in the brain which compensates for the spine maintenance deficits caused by a lack of E2 (Fig. 2*B*).

Although letrozole treatment did not affect spine density, it did reduce spine turnover rates, meaning that fewer spines were eliminated and added (Fig. 4). This form of structural plasticity is linked to functional plasticity (LTP and LTD): LTP induction in cultured hippocampal neurons increases spine turnover without affecting density (De Roo et al., 2008; Oe et al., 2013). Learning increases spine turnover in vivo in the neocortex, for example, in retrosplenial cortex during fear conditioning (Frank et al., 2018), during motor learning in motor cortex (Xu et al., 2009), and as a part of sensory-evoked plasticity (whisker stimulation) in somatosensory cortex (Alexander et al., 2018). This suggests that spine turnover (“remodeling”), rather than spine density, is the relevant correlate of plasticity. To that end, we wanted to test whether letrozole treatment, with its reduction in spine turnover, altered neocortical LTP. Indeed, we found that 4 d of letrozole treatment significantly reduced layer 4 to 3 theta burst stimulation evoked LTP in somatosensory cortex (Fig. 5). Consistent with this observation, letrozole treatment of ovariectomized mice abolishes hippocampal LTP (Vierk et al., 2012) and conversely, neuron-derived E2 has been shown to facilitate hippocampal LTP in acute slices (Tozzi et al., 2019). Testosterone, on the contrary, is involved in LTD induction (Di Mauro et al., 2017). Progesterone decreases LTP in hippocampal slices and does not affect LTD (Foy et al., 2008). Thus, the main steroid hormones do not have redundant functions in LTP/LTD, providing a plausible explanation how a lack of E2 regardless of other neurosteroids leads to an LTP reduction.

Reduced spine turnover and LTP might correlate with impaired cognition. We used the novel object recognition test to assess overall cognitive abilities of letrozole-treated mice (Fig. 6). Although we measured LTP and spine dynamics in somatosensory cortex, we should note that the novel object recognition test does not test functioning of somatosensory cortex but rather general cognitive abilities and hippocampal function. The results suggest that observed deficits might rely on common mechanisms outside of the brain areas we studied. On Day 4 of letrozole or vehicle treatment, animals explored two identical objects, and 24 h later one familiar and one novel object. Ovariectomy itself reduces performance in the novel object recognition test in rats (Wallace et al., 2006; Renczés et al., 2020), just as we observed in this study in mice. There is no overt difference between intact male and female rats (Abbott et al., 2016) or mice (Sierra et al., 2021). Prior reports show varying effects of letrozole, depending on the precise testing and administration protocol: letrozole infusion into dorsal hippocampus of ovariectomized mice directly after training leads to a worse performance in the test (Tuscher et al., 2016). Similarly, mice with a forebrain aromatase knock-out in excitatory neurons perform worse (Lu et al., 2019). On the contrary, lowering E2 and progesterone levels via hormonal contraceptives in rats did not affect their performance on the novel object and other cognitive/memory tests (Boi et al., 2022). Letrozole combined with androgen treatment did not affect test performance in rats (Luine et al., 2022) and adult ovariectomized rats significantly improved in the novel object recognition test when given androgens immediately following the baseline exploration (Frye and Lacey, 2001).

We confirmed that the performance of intact male and female control mice did not differ in our testing protocol and that ovariectomized mice performed significantly worse than intact controls. It is interesting to note that although the preference indices for letrozole-treated mice and male and female intact controls were not significantly different, naive female and male controls overall spent more total time exploring both objects than either group of ovariectomized mice (Fig. 6*B*). This indicates that E2 levels and aromatase inhibition affect additional behavioral parameters that were not captured with the novel object recognition test. Unexpectedly, letrozole treatment appeared to rescue the ovariectomy-induced deficit: letrozole-treated mice performed significantly better than vehicle-treated controls and were indistinguishable from intact female and male controls. This echoes the report on letrozole-treated ovariectomized rats (Aydin et al., 2008) in the Morris water maze test, where letrozole-treated animals performed better than vehicle-treated controls. We believe that these findings are consistent with a shift from estrogens to other steroid hormones upon aromatase inhibition and are supported by preclinical and clinical evidence: androgen supplementation in intact aged female mice enhances cognition (Benice and Raber, 2009), androgens acting on ERβ have anxiolytic and cognitive-enhancing effect (Frye et al., 2008), and women receiving aromatase inhibitors have better cognitive outcomes than those on SERMs (Phillips et al., 2010, 2011). Taken together, this study highlights the distinct roles or brain-derived versus gonadal E2 in plasticity and cognition, as well as the redundancy in some, but not all, functions of brain-derived E2.

## Synthesis

Reviewing Editor: Timothy Jarome, Virginia Polytechnic Institute and State University Decisions are customarily a result of the Reviewing Editor and the peer reviewers coming together and discussing their recommendations until a consensus is reached. When revisions are invited, a fact-based synthesis statement explaining their decision and outlining what is needed to prepare a revision will be listed below. The following reviewer(s) agreed to reveal their identity: NONE. Note: If this manuscript was transferred from JNeurosci and a decision was made to accept the manuscript without peer review, a brief statement to this effect will instead be what is listed below.

Both reviewers agreed that the manuscript is improved over the prior version and appreciate the edits that have already been made. However, several concerns remain that were raised during the original review which still need addressed before the manuscript can be considered for publication.

1) As mentioned before, aromatase antibodies are notoriously unreliable in mammalian brain tissue. The authors have provided no validation of the antibody they used, no corroborating evidence for aromatase expression using other methods, and the staining in Fig 2A looks non-specific (all cell bodies appear to be stained and background is high); the authors themselves refer to their immunostaining as "weak and variable." These points, together with the fact that other groups using different methods (e.g., Nirao Shah's work) do not find aromatase expression in somatosensory cortex of mice strongly suggest that the aromatase staining shown in Fig 2A artifactual. Without more compelling evidence for aromatase expression in somatosensory cortex, Fig 2A, statements about aromatase expression, and statements that letrozole likely affects somatosensory neurons directly should be removed.

2) The observation that letrozole decreases spine turnover in somatosensory cortex of ovariectomized mice is interesting. The longitudinal imaging approach is key to this finding. That said, it remains very difficult to appreciate the result based on Fig 3A because the few pale green "added" spines are barely distinguishable from the many pale blue "persistent" spines. Purple, orange, green, and white typically work very well as a four-color combination that allows all colors to be appreciated.

3) That letrozole appears to attenuate LTP in somatosensory cortex is also potentially interesting. However, the LTP experiments continue to ignore the substantial fraction of slices that showed a decrease in fEPSP amplitude (Fig 5E). In their response, the authors say, "we decided that by analyzing only the potentiating slices, we at least know for sure that these slices were healthy." How do they know this? Slice health is not a categorical feature: healthy or unhealthy; every slice in an in vitro electrophysiology experiment is the process of dying. Although the authors acknowledge that decreased fEPSP amplitude could reflect LTD, they do not investigate this possibility. They could have done so with data in-hand. For example, looking at the time course of each experiment would show, for each slice, whether the decrease in fEPEP amplitude appears promptly after theta burst stimulation (likely LTD), reflects a slow decline in amplitude throughout the experiment (likely pathological), or drops precipitously at the end of the experiment (also likely pathological but suggesting a different time range could be used for quantification). The possibility of LTD is relevant to the science of this study because androgens should be increased by letrozole treatment and androgens have been shown to promote LTD. Without exploring these possibilities, the authors leave a reader wondering about half the data that were excluded from analysis and may have missed an important result.

4) The authors have tempered their implication that performance in the object recognition test has anything to do with synaptic plasticity in the somatosensory cortex. That is good. However, it remains perplexing that an object recognition experiment is included in a paper about synaptic plasticity in the somatosensory cortex. The authors state in their response "Unfortunately, there are no good behavioral tests to assess somatosensory cortex function." That is not accurate. It is recommended that the object recognition experiment be removed from this paper as it focuses on the somatosensory cortex and should instead be part of another study.

5) Although they have expanded discussion as to how their results fit in with previous work regarding brain-derived estrogens, there is still an important gap. Some mention of the relationship between circulating and brain-derived estrogens and the potential impact of the length of ovariectomy on the production of brain-derived estrogens is warranted.
